# Precocious Preclinical Cardiovascular Sonographic Markers in Metabolically Healthy and Unhealthy Childhood Obesity

**DOI:** 10.3389/fendo.2020.00056

**Published:** 2020-03-03

**Authors:** Domenico Corica, Lilia Oreto, Giorgia Pepe, Maria Pia Calabrò, Luca Longobardo, Letteria Morabito, Giovanni Battista Pajno, Angela Alibrandi, Tommaso Aversa, Malgorzata Wasniewska

**Affiliations:** ^1^Department of Human Pathology of Adulthood and Childhood “G. Barresi”, University of Messina, Messina, Italy; ^2^Department of Clinical and Experimental Medicine, University of Messina, Messina, Italy; ^3^Department of Economics, University of Messina, Messina, Italy

**Keywords:** insulin resistance, global longitudinal strain, epicardial adipose tissue, cardiometabolic risk, myocardial dysfunction

## Abstract

**Background:** Childhood obesity is related to a wide spectrum of cardiovascular and metabolic comorbidities.

**Objectives:** (1) To identify precocious, preclinical, cardiovascular sonographic modifications, in a cohort of overweight (OW) and obese (OB) children and adolescents compared to lean controls; (2) to investigate the association between clinical and metabolic variables and cardiovascular sonographic parameters; (3) to evaluate their relation with two different phenotypes of obesity: metabolically healthy obesity (MHO) and metabolically unhealthy obesity (MUO).

**Materials and Methods:** Fifty-nine OW and OB children and adolescents (9.8 ± 2.9 years) and 20 matched lean controls underwent anthropometric, biochemical, echocardiography assessment, and sonographic evaluation of carotid artery and ascending aorta (AA). OW and OB subjects were divided in MHO and MUO, according to the Camhi et al. definition.

**Results:** OW and OB children showed significantly higher left ventricular (LV) dimensions and mass, carotid artery intima–media thickness (CIMT), carotid stiffness [β-index, pulse wave velocity (PWV)], significantly lower mitral peak early (E) and late (A) velocity ratio (E/A ratio), and significantly impaired global longitudinal strain (GLS) compared to controls. BMI SD and HOMA-IR were positively significantly related to LV dimensions, LA volume and epicardial adipose tissue (EAT), and negative to E/A ratio. Waist circumference (WC) was positively correlated to LV dimensions, LA volume, CIMT, PWV, AA diameter, and EAT. Furthermore, WC was a strong predictor of LV dimensions, LA volume and strain, AA stiffness and diameter; BMI SD was significantly associated with EAT, LVM index, and E/A ratio; HOMA-IR and triglycerides were significant predictors of GLS. MUO patients showed higher BMI SD (*p* = 0.02), WC (*p* = 0.001), WHtR (*p* = 0.001), HOMA-IR (*p* = 0.004), triglycerides (*p* = 0.01), SBP (*p* = 0.001), as well as LV dimensions, EAT (*p* = 0.03), CIMT (*p* = 0.01), AA diameter (*p* = 0.02), β-index (*p* = 0.03) and PWV (*p* = 0.002), AA stiffness (*p* = 0.006), and significantly impaired GLS (*p* = 0.042) compared to MHO.

**Conclusions:** Severity of overweight, abdominal obesity, insulin resistance, and MUO phenotype negatively affect cardiovascular remodeling and subclinical myocardial dysfunction in OW and OB children. MUO phenotype is likely to increase the risk of developing cardiometabolic complications since the pediatric age. Distinction between MHO and MUO phenotypes might be useful in planning a personalized follow-up approach in obese children.

## Introduction

Obesity in childhood is related to a wide spectrum of cardiovascular and metabolic comorbidities. Obese children and adolescents are more likely to become obese adults, with an increased risk of premature morbidity and mortality due to cardiovascular diseases (CVD) (1–5). The presence of early signs of cardiovascular (CV) dysfunction have already been demonstrated in obese children and adolescents, even in absence of other obese-related comorbidities, as insulin resistance, dyslipidemia, and arterial hypertension (6).

A categorization of obesity in two different phenotypes have been proposed. Accordingly, it is possible to distinguish the metabolically unhealthy obesity (MUO), characterized by “unfavorable” cardiometabolic profile, and the metabolically healthy obesity (MHO), with “favorable” lipid, glycemic, and blood pressure profiles (7). To date, health and clinical implications of this distinction remain controversial. Several prospective studies reported a lower risk of CVD in MHO subjects in comparison to MUO, without demonstrating an increased risk of CVD when compared with the general population (8, 9). On the contrary, other studies documented a long-term increased risk of CVD and early obesity-related complications also in MHO subjects (10, 11). Moreover, the absence of univocal diagnostic criteria to define MUO makes it difficult to compare both adulthood and childhood studies (7). Interestingly, a shift from MHO to MUO seems to occur more frequently during transition from adolescence to adulthood (12); however, less is known about the clinical implications of MUO phenotype in childhood.

Structural and functional cardiovascular modifications, such as left ventricle hypertrophy, systolic/diastolic dysfunction, increased carotid intima–media thickness(CIMT), have been considered preclinical indices of CVD in obese adults (13), as well as in pediatric obese subjects (14, 15). In this context, two parameters in obese patients' echocardiographic assessment became increasingly important: the left ventricular (LV) global longitudinal strain (GLS) and the epicardial adipose tissue (EAT). GLS is considered a reliable and reproducible parameter for the assessment of myocardial contractility (16). Strain imaging is able to early detect subclinical myocardial abnormalities in subjects affected by cardiovascular and metabolic diseases, showing a better diagnostic efficacy and prognostic value for predicting cardiovascular events compared to LV ejection fraction (EF) (17, 18). EAT is a metabolically active adipose tissue localized around the heart, between the myocardium and the visceral layers of the pericardium (19), that is strictly related to visceral and subcutaneous fat (19) and to the pathogenesis of the CVD associated with obesity (20).

Relationships between precocious cardiovascular changes and metabolic alterations are not widely investigated in children.

In light of these pieces of evidence, the aims of this study are (1) to identify precocious, preclinical, cardiovascular, structural, and functional sonographic modifications, in a cohort of overweight (OW) and obese (OB) children and adolescents; (2) to investigate the association between clinical and metabolic variables and cardiovascular sonographic parameters; (3) to evaluate their relation with two different phenotypes of obesity: MHO and MUO.

## Materials and Methods

### Study Design and Population

This is a single-center, cross-sectional, case-control study carried out at the Pediatric Endocrinology Outpatient Clinic at the University of Messina, Italy, during a period of 6 months (from September 2017 to March 2018). Fifty-nine Caucasian OW and OB children and adolescents were brought to the Outpatient Clinic for first evaluation, and 20 age- and sex-matched, lean (BMI SD ≤ 1) controls, were consecutively recruited. The inclusion criteria were BMI > 1 SD according to the WHO definition (21), age range between 5 and 16 years, Caucasian ethnicity, and born as healthy full-term infant adequate for gestational age. The exclusion criteria were genetic and/or endocrine causes of obesity, diabetes, CVD, either pre-term or post-term birth, chronic diseases, chronic pharmacological therapies, and smoking.

### Clinical Evaluation and Laboratory Assessment

Detailed history from the parents and from clinical records was obtained. At recruitment, physical evaluation was performed according to standardized procedures, as previously described (22). Body weight was measured to the nearest 0.1 kg on accurate and properly calibrated standard beam scales, in minimal underclothes and no shoes. Height was measured to the nearest 0.1 cm on standardized, wall-mounted height boards, according to standardized procedures. The children stood with the head aligned in the Frankfort plane, barefoot, with feet placed together and flat on the ground, heels, buttocks, and scapulae against the vertical backboard, arms loose and relaxed with the palms facing medially. BMI was calculated using the equation: body weight (kg)/height (m)^2^. BMI values were standardized using age- and sex-specific standard deviation (SD) based on WHO growth references (21). Waist circumference (WC) was measured, to the nearest 0.5 cm while the subjects were standing, after gently exhaling, as the minimal circumference measurable on the horizontal plane between the lowest portion of the rib cage and the iliac crest (23). Waist-to-height ratio (WHtR), an index of body fat distribution, was calculated as previously described (24).

Patients underwent a detailed physical examination and pubertal evaluation, assessed by five Tanner stages of breast development in girls and testicular volume in boys (25), performed by pediatric endocrinologists. Pubertal stage was defined from G2 or B2 to G5 or B5 Tanner's stages (25).

Systolic blood pressure (SBP) and diastolic blood pressure (DBP) were recorded at rest three times on the right arm in mmHg using a manual sphygmomanometer; for analysis, the average of three blood pressure values was used (26).

A fasting blood sampling for plasma triglycerides, high-density lipoproteins (HDL), low-density lipoproteins (LDL), total cholesterol, glucose and insulin was performed at least 8 h after the last meal. These parameters were analyzed with standard techniques: triglycerides were measured enzymatically, the HDL-cholesterol fraction was obtained after precipitation using a phosphotungstic reagent, and glucose was measured using a glucose oxidase method; serum insulin was determined by a chemiluminescence immunoassay. Thyroid, liver, and kidney function tests were also performed. Oral glucose tolerance test (OGTT) was performed with the standard method (1.75 g/kg of body weight, up to a maximum of 75 g) in OW and OB patients only, measuring glucose and insulin serum levels at baseline and during OGTT (at 0, 30, 60, 90, and 120 min), and impaired glucose tolerance or type 2 diabetes were diagnosed according to the criteria of the American Diabetes Association (27). Insulin resistance was measured through homeostasis model assessment of insulin resistance (HOMA-IR). This index was calculated using the equation: Fasting insulin (μU/ml) × Fasting glucose (mg/dl)/405 (28). Insulin resistance was defined as a HOMA-IR > 2.5 in prepubertal children and >4 in pubertal subjects (29).

Among OW and OB patients, MUO was defined as the presence of two or more of the following cardiometabolic risk factors (12): triglycerides ≥ 110 mg/dl or on cholesterol medication; HDL <40 mg/dl or on cholesterol medication; fasting glucose ≥ 100 mg/dl or on glucose/insulin medication; blood pressure ≥ 90th percentile for age, gender, and height or on blood pressure medication.

### Echocardiographic Conventional Parameters

All participants underwent 2D transthoracic echocardiography (TTE) with Vivid E95 echocardiography equipment (GE Vingmed Ultrasound AS, Horten, Norway). Image acquisition was performed at a frame rate of 70–90 frames per second, and three cardiac cycles were stored in cine loop format for subsequent off-line calculation using a specific software workstation (EchoPAC version 7.0.0; GE Vingmed Ultrasound AS). All the echocardiographic measurements were obtained according to the current guidelines (30, 31). Linear internal measurements of the LV and its walls were performed in the parasternal long-axis view. Specifically, interventricular septum (IVSD), diastolic left ventricle posterior wall (PWD), end-diastolic (LVEDD) and end-systolic (LVESD) LV diameters, were evaluated. Using these measurements, LV mass was automatically calculated; therefore, LV mass was divided to height (meters) to the power of 2.7 to obtain the LV mass index (LVM-index) (32). LV volumes and EF were calculated by the Simpson method in the apical three-, four-, and two-chamber views. Similarly, the left atrial (LA) volume was calculated in the apical four- and tow-chamber views by the method of disks. EAT was measured as the echo-free space between the outer wall of the myocardium and the visceral layer of the pericardium perpendicularly to the free wall of the right ventricle at end systole in the parasternal long-axis view. Ascending aorta (AA) diameters were calculated from the parasternal long-axis view at the maximal diameter of the sinuses of Valsalva, in systole (AoS) and in diastole (AoD). AA stiffness was calculated by the following formula: (ln (SBP/DBP)/[(AoS – AoD)/AoD]) (33). Mitral peak early (E) and late (A) velocities, and E/A ratio, were obtained by pulsed-wave Doppler performed in the apical four-chamber view, placing the sample volume at the tip of mitral leaflets; septal and lateral early diastolic mitral annular velocities (E′) were evaluated by pulsed-wave tissue Doppler imaging (TDI), and the ratio between mitral peak early velocity and the averaged value of septal and lateral early diastolic mitral annular velocities (E/E′) was calculated; the maximum tricuspid regurgitation (TR) velocity was measured in the apical four-chamber view through continuous wave Doppler.

### 2D Speckle Tracking Echocardiography Analysis and Evaluation of Carotid Artery Stiffness

For the evaluation of LV 2D speckle tracking, GLS images were obtained from the apical four-, three-, and two-chamber views. Using a customized commercial speckle-tracking software (EchoPAC version 7.0.0; GE Vingmed Ultrasound AS), LV GLS was calculated placing fiducial landmarks to define the base and apex of LV; the software automatically generated the region of interest and, after the processing, the bull's-eye maps that allowed the calculation of the averaged value of GLS (30). Using the same software, the LA endocardium surface was manually traced in the four- and two-chamber views by a point-and-click approach, excluding the appendage and pulmonary veins. An epicardial surface tracing was then automatically generated by the system, and the region of interest, divided into six segments, was identified. The software generates the longitudinal strain curves for each of these segments, together with a mean curve of all segments, whose maximal positive peak was used to calculate the LA reservoir strain value. The LA strain was determined as the average value from all segments of the LA in the apical four-chamber and two-chamber views (30).

The carotid ultrasound examinations were performed using a color Doppler echocardiography machine (Prosound Alpha 10, Aloka, Tokyo, Japan) equipped with a 7.5-MHz linear array probe high-resolution echo-tracking system that allows accurate measurements of carotid diameter changes. Pressure waveforms, calibrated on systolic and diastolic blood pressure values measured with a cuff-type manometer applied to the right upper arm, were non-invasively obtained using arterial diameter change (systolic–diastolic diameter). Validated parameters of arterial stiffness [β-index, pulse wave velocity (PWV) and augmentation index] were automatically calculated as a mean of five beats, as already reported (31, 34). Moreover, CIMT, defined as the distance between the lumen/intima and the media/adventitia interfaces, was evaluated. All measurements were taken manually at the far wall of the vessel from perfectly horizontal images of distal carotid common artery (about 1.5 cm proximal to the carotid bifurcation), in longitudinal planes, with a transducer depth of 4 cm and from a posterolateral approach.

### Statistical Analysis

The numerical data were expressed as the mean and standard deviations (SD) while the categorical variables as number and percentage. The non-parametric approach was used due to sample size dimension and since not all numerical variables (prevalently metabolic variables) were normally distributed, as verified by the Kolmogorov–Smirnov test. The Mann–Whitney test was applied in order to compare cases and healthy controls with reference to anthropometric and cardiovascular parameters; the Chi Square test was applied in order to compare these groups with reference to categorical variables. Moreover, the Mann–Whitney test was performed, within cases, to evaluate possible differences between patients with or without insulin resistance and between MUO or MHO subjects. The Spearman correlation test was applied to assess the existence of any significant interdependence between numerical parameters. Two stepwise multivariable linear regression models were estimated in order to individuate the most significant predictors of each cardiovascular parameter (IVSD, PWD, LVEDD, LVSED, LVM index, LA volume, LA strain, E/A ratio, E′, E/E′ ratio, GLS, CIMT, PWV, β-index, Augmentation index, AA diameter, AA stiffness, EAT) according to the following models: Model 1 (age, gender, pubertal stage, BMI SD, WC, HOMA-IR, LDL, HDL, triglycerides, SBP, duration of obesity) and Model 2 (age, gender, pubertal stage, BMI SD, WHtR, HOMA-IR, LDL, HDL, triglycerides, SBP, duration of obesity). The normal distribution shown by the ultrasound parameters provides the methodological guarantee for the use of linear regression models. A *p* < 0.050 was considered to be statistically significant. Statistical analyses were performed using SPSS for Window package, version 22.

## Results

### Clinical Characteristics and Biochemical Evaluation

Seventy-nine children and adolescents were consecutively recruited: 59 OW and OB were included in group A, while 20 lean controls were included in group B. Groups A and B were comparable for age (9.8 ± 2.9 vs. 8.6 ± 2.9; *p* = 0.07), gender (33 males/26 females vs. 10/10; *p* = 0.65), and pubertal stage (30 pre-pubertal/29 pubertal vs. 12/8; *p* = 0.48). All subjects of group A presented with abdominal obesity (WHtR ≥ 0.5; 0.61 ± 0.05). Thyroid, liver, and kidney function tests were normal in the entire population (data not shown). Insulin resistance was observed in 47.5% of group A patients. OGTT documented a condition of impaired glucose tolerance in four patients of group A (6.8%) who had normal fasting glucose; diabetes was excluded in the entire cohort. OW and OB children showed significantly higher SBP compared to controls, although in seven patients of group A (11.8%), an above maximum of range SBP value was documented, according to the Flynn et al. criteria (26).

### Cardiovascular Assessment

Comparison analysis documented a significantly higher IVSD (7.8 ± 1.1 vs. 7 ± 1.2 mm; *p* = 0.006), PWD (7.8 ± 1.3 vs. 5.9 ± 0.7 mm; *p* = 0.000), LVEDD (43.5 ± 4.6 vs. 36.9 ± 5.4 mm; *p* = 0.000), LVESD (27.3 ± 3.2 vs. 22.6 ± 3.8 mm; *p* = 0.000), LVM-index (37 ± 7.2 vs. 32.1 ± 9; *p* = 0.006), CIMT (4.9 ± 0.8 vs. 3.3 ± 0.3 mm; *p* = 0.000), β-index (3.2 ± 0.8 vs. 2.7 ± 0.4; *p* = 0.007), PWV (3.7 ± 0.5 vs. 3.3 ± 0.3 m/s; *p* = 0.004) in group A compared to group B (Table 1). Moreover, the E/A ratio (1.9 ± 0.4 vs. 2.2 ± 0.5; *p* = 0.003) and EF (67.3 ± 3.7 vs. 69.7 ± 4.0%; *p* = 0.041) were significantly lower, and GLS was significantly impaired (−18.7 ± 2.2 vs. −23.9 ± 2.4%; *p* = 0.000) in OW and OB subjects compared to controls.

**Table 1:** Significant differences between OW and OB children (Group A) and controls (Group B).

	**Group A (*n* = 59)**	**Group B (*n* = 20)**	***P***
BMI SD	2.2 ± 0.5	−0.3 ± 0.8	0.000
SBP (mmHg)	112.5 ± 13.4	103 ± 7.3	0.006
IVSD (mm)	7.8 ± 1.1	7 ± 1.2	0.006
PWD (mm)	7.8 ± 1.3	5.9 ± 0.7	0.000
LVEDD (mm)	43.5 ± 4.6	36.9 ± 5.4	0.000
LVESD (mm)	27.3 ± 3.2	22.6 ± 3.8	0.000
LVM-index	37 ± 7.2	32.1 ± 9	0.006
EF (%)	67.3 ± 3.7	69.7 ± 4	0.041
E/A ratio	1.9 ± 0.4	2.2 ± 0.5	0.003
GLS (%)	−18.7 ± 2.2	−23.9 ± 2.4	0.000
CIMT (mm)	4.9 ± 0.8	3.3 ± 0.3	0.000
β-index	3.2 ± 0.8	2.7 ± 0.4	0.007
PWV (m/s)	3.7 ± 0.5	3.3 ± 0.3	0.004

Numerical data are expressed as mean ± SD.

*BMI SD, body mass index standard deviation; SBP, systolic blood pressure; IVSD, interventricular septum; PWD, diastolic left ventricle posterior wall; LVEDD, end-diastolic; LVESD, end-systolic, left ventricular diameters; LVM-index, left ventricle mass index; E/A ratio, early (E) and late (A) mitral peak velocities ratio; GLS, global longitudinal strain; CIMT, carotid intima–media thickness; PWV, pulse wave velocity*.

Comparison between group A patients with and without insulin resistance are reported in Table 2.

**Table 2:** Comparison analysis between patients of group A with and without insulin resistance.

	**Non-IR patients** **(*n* = 31)**	**IR patients** **(*n* = 28)**	***p***
WC (cm)	87.6 ± 12.8	95.4 ± 13.5	**0.03**
WHtR	0.60 ± 0.05	0.64 ± 0.05	**0.03**
BMI SD	2.1 ± 0.4	2.4 ± 0.5	**0.02**
SBP (mmHg)	111.4 ± 14.1	113.8 ± 12.7	0.39
DBP (mmHg)	68.2 ± 11.3	67.3 ± 9.4	0.42
IVSD (mm)	7.7 ± 1.1	7.9 ± 1.2	0.48
PWD (mm)	7.7 ± 1.3	7.8 ± 1.2	0.69
LVEDD (mm)	42.7 ± 4.6	44.34.5	0.34
LVESD (mm)	26.8 ± 3.2	27.9 ± 3	0.23
LVM-index	35.8 ± 5.8	38.5 ± 8.4	0.28
LA volume (ml)	40.8 ± 11.4	42.6 ± 11.7	0.61
LA strain (%)	40.5 ± 9.9	39.3 ± 10.8	0.54
EF (%)	67.5 ± 4.1	67.2 ± 3.2	0.74
E/A ratio	1.9 ± 0.5	1.8 ± 0.4	0.66
E′ (cm/s)	15.8 ± 2.1	14.9 ± 2.6	0.25
E/E′ ratio	6.3 ± 0.9	6.5 ± 1.1	0.28
GLS (%)	−19.5 ± 1.4	−17.8 ± 2.4	**0.03**
EAT (mm)	12.6 ± 2.1	13.3 ± 2.6	0.43
CIMT (mm)	4.8 ± 0.8	4.9 ± 0.7	0.59
β-index	3.1 ± 0.9	3.3 ± 0.7	0.38
PWV (m/s)	3.6 ± 0.6	3.8 ± 0.4	0.25
Augmentation index	4.4 ± 13.1	5.1 ± 15.4	0.93
AA diameter (mm)	23.4 ± 3.2	22.4 ± 2.8	0.33
AA stiffness	2.4 ± 0.4	2.7 ± 0.4	**0.003**

Numerical data are express as mean ± SD.

Insulin resistance was defined as a HOMA-IR > 2.5 in pre-pubertal and >4 in pubertal subjects.

IR, Insulin resistant; HOMA-IR, homeostasis model assessment of insulin resistance; WC, waist circumference; WHtR, waist-to-height ratio; BMI SD, body mass index standard deviation; SBP, systolic blood pressure; DBP, diastolic blood pressure; IVSD, interventricular septum; PWD, diastolic left ventricle posterior wall; LVEDD, end-diastolic left ventricular diameter; LVESD, end-systolic left ventricular diameter; LVM-index, left ventricle mass index; LA, left atrial; E/A ratio, early (E) and late (A) mitral peak velocities ratio; E′, early diastolic mitral annular velocities; E/E′, septal and lateral early diastolic mitral annular velocities; GLS, global longitudinal strain; CIMT, carotid intima–media thickness; PWV, pulse wave velocity; AA, ascending aorta; EAT, epicardial adipose tissue.

*Bold values indicate a statistically significant p-value*.

Results of correlation analysis are reported in Table 3. In particular, BMI SD and HOMA-IR were positively significantly related to LV dimensions, LA volume, and EAT, and negatively to E/A ratio. WC was positively correlated to SBP, DBP, LV dimensions, LA volume, E/E′ ratio, CIMT, PWV, AA diameter, EAT, and negatively with LA strain. Moreover, EAT was positively significantly related to LV dimensions, LA volume, SBP, CIMT, AA diameter and negatively with LA strain.

**Table 3:** Bivariate correlation analysis.

		**BMI SD**	**HOMA-IR**	**SBP**	**WC**	**WHtR**	**EAT**
SBP	*r*	0.340	0.340	–	0.569	0.428	0.489
	*p*	**0.008**	**0.008**		**0.000**	**0.004**	**0.002**
DBP	*r*	0.099	0.225	–	0.375	0.282	−0.074
	*p*	0.456	0.087		**0.012**	0.064	0.665
IVSD	*r*	0.260	0.343	0.353	0.573	0.450	0.558
	*p*	**0.044**	**0.008**	**0.006**	**0.000**	**0.002**	**0.000**
PWD	*r*	0.323	0.222	0.282	0.517	0.423	0.706
	*p*	**0.012**	0.091	**0.030**	**0.000**	**0.004**	**0.000**
LVEDD	*r*	0.143	0.268	0.359	0.387	0.099	0.577
	*p*	0.280	**0.040**	**0.005**	**0.009**	0.524	**0.000**
LVESD	*r*	0.190	0.322	0.361	0.418	0.121	0.500
	*p*	0.150	**0.013**	**0.005**	**0.005**	0.432	**0.002**
EF	*r*	−0.022	−0.058	−0.021	−0.101	−0.044	0.020
	*p*	0.866	0.663	0.873	0.515	0.775	0.908
LVM-index	*r*	0.158	0.008	−0.07	−0.157	0.075	0.323
	*p*	0.244	0.954	0.61	0.326	0.640	0.055
LA volume	*r*	0.303	0.277	0.363	0.593	0.237	0.525
	*p*	**0.026**	**0.043**	**0.007**	**0.000**	0.136	**0.002**
LA strain	*r*	−0.101	−0.434	−0.352	−0.619	−0.235	−0.387
	*p*	0.571	**0.010**	**0.041**	**0.002**	0.291	**0.029**
E/A ratio	*r*	−0.407	−0.129	−0.001	−0.173	−0.361	−0.236
	*p*	**0.001**	0.331	0.994	0.262	**0.016**	0.159
E′	*r*	−0.140	−0.284	−0.257	−0.460	−0.297	−0.327
	*p*	0.342	***0.051***	0.078	**0.006**	0.088	0.067
E/E′ ratio	*r*	−0.019	0.242	0.313	0.487	0.387	0.227
	*p*	0.900	0.098	**0.030**	**0.003**	**0.024**	0.211
GLS	*r*	−0.161	−0.314	−0.389	0.176	0.195	0.228
	*p*	0.355	0.066	**0.021**	0.422	0.372	0.210
CIMT	*r*	0.157	0.027	−0.006	0.242	0.330	0.441
	*p*	0.249	0.842	0.966	0.122	**0.033**	**0.008**
β-index	*r*	0.140	0.133	0.327	0.221	0.178	0.323
	*p*	0.298	0.323	**0.013**	0.155	0.253	0.055
PWV	*r*	0.252	0.283	0.644	0.472	0.383	0.294
	*p*	0.059	**0.033**	**0.0001**	**0.001**	**0.011**	0.081
Augmentation index	*r*	−0.137	−0.13	0.022	0.025	0.009	0.159
	*p*	0.315	0.923	0.874	0.873	0.956	0.362
AA diameter	*r*	0.087	0.238	0.267	0.554	0.275	0.465
	*p*	0.631	0.182	0.133	**0.011**	0.241	**0.006**
AA stiffness	*r*	−0.057	0.446	0.075	0.296	0.178	0.213
	*p*	0.753	**0.009**	0.680	0.205	0.453	0.233
EAT	*r*	0.431	0.349	0.489	0.519	0.396	–
	*p*	**0.008**	**0.034**	**0.002**	**0.011**	0.062	

IR, Insulin resistant; HOMA-IR, homeostasis model assessment of insulin resistance; WC, waist circumference; WHtR, waist-to-height ratio; BMI SD, body mass index standard deviation; SBP, systolic blood pressure; DBP, diastolic blood pressure; IVSD, interventricular septum; PWD, diastolic left ventricle posterior wall; LVEDD, end-diastolic left ventricular diameter; LVESD, end-systolic left ventricular diameter; LVM-index, left ventricle mass index; LA, left atrial; E/A ratio, early (E) and late (A) mitral peak velocities ratio; E′, early diastolic mitral annular velocities; E/E′, septal and lateral early diastolic mitral annular velocities; GLS, global longitudinal strain; CIMT, carotid intima–media thickness; PWV, pulse wave velocity; AA, ascending aorta; EAT, epicardial adipose tissue.

*Bold values indicate a statistically significant p-value*.

To investigate the independent effect of anthropometric and biochemical variables on cardiovascular parameters, two multivariate stepwise regression analyses were performed according to model 1 (Table 4) and model 2 (Table 5). WCs were very strong predictors of LV dimensions, LA volume and strain, AA stiffness and diameter (model 1). BMI SD was significantly associated with EAT, LVM-index, and E/A ratio (models 1 and 2). HOMA-IR and triglycerides were significant predictors of GLS (models 1 and 2).

**Table 4:** Stepwise multivariate linear regression analysis for WC, BMI SD, HOMA-IR, LDL, HDL, triglycerides, SBP, obesity duration, age, sex, and pubertal stage (model 1).

**Predictors**	**B**	**SE**	***p*-value**
**IVSD**			
WC	0.036	0.012	0.005
**PWD**			
WC	0.034	0.015	0.03
**LVEDD**			
WC	0.126	0.041	0.004
Triglycerides	0.042	0.016	0.013
**LVESD**			
WC	0.110	0.030	0.001
**LVM-index**			
BMI SD	7.016	2.968	0.023
**LA volume**			
WC	0.503	0.108	0.000
**LA strain**			
WC	−0.522	0.143	0.002
**E/A ratio**			
BMI SD	−0.391	0.139	0.008
**E′**			
WC	−0.095	0.025	0.001
**E/E′ ratio**			
HOMA-IR	0.218	0.073	0.005
**GLS**			
HOMA-IR	0.780	0.285	0.013
Triglycerides	0.022	0.010	0.042
**CIMT**			
BMI SD	0.678	0.242	0.008
**PWV**			
SBP	0.020	0.004	0.000
HDL	−0.007	0.004	0.041
**AA diameter**			
WC	0.131	0.038	0.003
**AA Stiffness**			
WC	0.017	0.006	0.010
**EAT**			
BMI SD	3.744	0.976	0.001

*B, regression coefficient; SE, standard error; IR, Insulin resistant; HOMA-IR, homeostasis model assessment of insulin resistance; WC, waist circumference; WHtR, waist-to-height ratio; BMI SD, body mass index standard deviation; SBP, systolic blood pressure; DBP, diastolic blood pressure; IVSD, interventricular septum; PWD, diastolic left ventricle posterior wall; LVEDD, end-diastolic left ventricular diameter; LVESD, end-systolic left ventricular diameter; LVM-index, left ventricle mass index; LA, left atrial; E/A ratio, early (E) and late (A) mitral peak velocities ratio; E′, early diastolic mitral annular velocities; E/E′, septal and lateral early diastolic mitral annular velocities; GLS, global longitudinal strain; CIMT, carotid intima–media thickness; PWV, pulse wave velocity; AA, ascending aorta; EAT, epicardial adipose tissue*.

**Table 5:** Stepwise multivariate linear regression analysis for WHtR, BMI SD, HOMA-IR, LDL, HDL, triglycerides, SBP, obesity duration, age, sex, and pubertal stage (model 2).

**Predictors**	**B**	**SE**	***p*-value**
**IVSD**			
BMI SD	0.749	0.322	0.02
**PWD**			
BMI SD	1.478	0.352	0.000
**LVEDD**			
HOMA-IR	0.459	0.218	0.042
Triglycerides	0.046	0.016	0.07
Obesity duration	0.333	0.147	0.03
**LVESD**			
HOMA-IR	0.343	0.166	0.043
Triglycerides	0.025	0.012	0.042
Obesity duration	0.230	0.112	0.041
**LVM-index**			
BMI SD	7.016	2.968	0.023
**LA volume**			
BMI SD	9.802	3.573	0.009
**LA strain**			
HOMA-IR	−3.247	1.234	0.016
**E/A ratio**			
BMI SD	−0.391	0.139	0.008
**E′**			
HOMA-IR	−0.670	0.188	0.001
**E/E′ ratio**			
HOMA-IR	0.218	0.073	0.005
**GLS**			
HOMA-IR	0.780	0.285	0.013
Triglycerides	0.022	0.010	0.042
**CIMT**			
WHtR	6.436	1.997	0.003
**PWV**			
SBP	0.020	0.004	0.000
HDL	−0.007	0.004	0.041
**AA diameter**			
BMI SD	3.245	1.248	0.019
Obesity duration	0.319	0.130	0.026
**AA Stiffness**			
SBP	0.014	0.006	0.026
LDL	0.007	0.003	0.021
**EAT**			
BMI SD	3.744	0.976	0.001

*B, regression coefficient; SE, standard error; IR, Insulin resistant; HOMA-IR, homeostasis model assessment of insulin resistance; WC, waist circumference; WHtR, waist-to-height ratio; BMI SD, body mass index standard deviation; SBP, systolic blood pressure; DBP, diastolic blood pressure; IVSD, interventricular septum; PWD, diastolic left ventricle posterior wall; LVEDD, end-diastolic left ventricular diameter; LVESD, end-systolic left ventricular diameter; LVM-index, left ventricle mass index; LA, left atrial; E/A ratio, early (E) and late (A) mitral peak velocities ratio; E′, early diastolic mitral annular velocities; E/E′, septal and lateral early diastolic mitral annular velocities; GLS, global longitudinal strain; CIMT, carotid intima–media thickness; PWV, pulse wave velocity; AA, ascending aorta; EAT, epicardial adipose tissue*.

### MUO vs. MHO: Clinical, Biochemical, Cardiovascular Evaluation

Thirteen OW and OB patients (22%) were classified as MUO, according to the Camhi et al. criteria (12). MUO patients showed significantly higher BMI SD, WC, WHtR, HOMA-IR, triglycerides, SBP, as well as LV dimensions, EAT, CIMT, AA diameter, carotid, and AA stiffness compared to MHO patients (Table 6). Moreover, GLS was significantly impaired in MUO (Table 6).

**Table 6:** Comparison analysis between MUO and MHO phenotypes in group A.

	**MUO (*n* = 13)**	**MHO (*n* = 46)**	***p***
Age (years)	12.1 ± 2.8	10.7 ± 2.9	0.13
BMI SD	2.5 ± 0.6	2.1 ± 0.4	**0.02**
WC (cm)	103.3 ± 13.5	87.4 ± 11.2	**0.001**
WHtR	0.7 ± 0.1	0.6 ± 0.01	**0.001**
HOMA-IR	4.6 ± 2	3.1 ± 2.1	**0.004**
Total cholesterol (mg/dl)	166.8 ± 33.8	171.6 ± 26.5	0.79
HDL (mg/dl)	43.9 ± 7.4	53.5 ± 14	**0.002**
LDL (mg/dl)	98.2 ± 33.2	94.8 ± 27.9	0.87
Triglycerides(mg/dl)	105.9 ± 46.8	74 ± 27.7	**0.01**
GOT (U/l)	22.5 ± 5.4	21 ± 5.5	0.49
GPT (U/l)	31 ± 23	21.3 ± 7.1	0.19
PCR (mg/dl)	0.7 ± 1.5	0.3 ± 0.3	0.85
SBP (mmHg)	124.8 ± 14.7	109.1 ± 10.8	**0.001**
DBP (mmHg)	73.1 ± 12.7	66.3 ± 9.3	0.12
IVSD (mm)	8.4 ± 1.3	7.7 ± 1.1	**0.07**
PWD (mm)	8.4 ± 1.2	7.6 ± 1.3	**0.06**
LVEDD (mm)	46.3 ± 5.6	42.7 ± 4	**0.043**
LVESD (mm)	29.3 ± 3.7	26.8 ± 2.8	**0.02**
EF (%)	66.5 ± 3.3	67.6 ± 3.8	0.47
LVM-index	37.1 ± 7.2	36.5 ± 6.4	0.71
LA volume (ml)	45.4 ± 11.9	40.5 ± 11.3	0.26
LA strain (%)	37.6 ± 10.9	40.8 ± 10.1	0.48
E/A ratio	1.9 ± 0.4	1.9 ± 0.4	0.85
E′ (cm/s)	13.5 ± 1.9	15.9 ± 2.3	**0.004**
E/E′ ratio	7.2 ± 0.8	6.2 ± 1	**0.004**
GLS (%)	−17.3 ± 2.1	−19.1 ± 2	**0.042**
EAT (mm)	14.6 ± 1.7	12.6 ± 2.3	**0.03**
CIMT (mm)	5.5 ± 1.1	4.7 ± 0.8	**0.01**
β-index	3.6 ± 0.6	3.1 ± 0.9	**0.03**
PWV (m/s)	4.1 ± 0.3	3.6 ± 0.5	**0.002**
Augmentation index	0.1 ± 15.7	4.1 ± 19.1	0.33
AA diameter (mm)	25 ± 2	22.3 ± 3	**0.02**
AA stiffness	3 ± 0.6	2.4 ± 0.3	**0.006**

Numerical data are expressed as mean ± SD.

*MHO, metabolically healthy obesity; MUO, metabolically unhealthy childhood obesity; HOMA-IR, homeostasis model assessment of insulin resistance; WC, waist circumference; WHtR, waist-to-height ratio; BMI SD, body mass index standard deviation; SBP, systolic blood pressure; DBP, diastolic blood pressure; IVSD, interventricular septum; PWD, diastolic left ventricle posterior wall; LVEDD, end-diastolic left ventricular diameter; LVESD, end-systolic left ventricular diameter; LVM-index, left ventricle mass index; LA, left atrial; E/A ratio, early (E) and late (A) mitral peak velocities ratio; E′, early diastolic mitral annular velocities; E/E′, septal and lateral early diastolic mitral annular velocities; GLS, global longitudinal strain; CIMT, carotid intima–media thickness; PWV, pulse wave velocity; AA, ascending aorta; EAT, epicardial adipose tissue. Bold values indicate a statistically significant p-value*.

## Discussion

The present study demonstrated a negative effect of childhood obesity on subclinical structural and functional cardiovascular modifications. Particularly, severity of overweight, abdominal obesity, and insulin resistance were the main predictors of cardiovascular remodeling, subclinical myocardial dysfunction, and amount of EAT. MUO patients seem to have a significant unfavorable cardiometabolic profile.

### Systolic Myocardial Function and Myocardial Geometry

Evaluation of GLS has assumed increasing importance in LV systolic function assessment as a reliable and reproducible index of myocardial contractility. GLS is able to early identify subclinical myocardial abnormalities differently from EF that is characterized by intrinsic limitations, as late reduction only in an advanced stage of CVD, poor reliability in patients with LV hypertrophy and volume reduction, inter-observer and intra-observer variability due to apical foreshortening (16). Accordingly, in our study EF did not differ between groups.

In our cohort, OW and OB children showed a significantly impaired GLS compared to controls, that is, expression of a decreased LV myocardial deformation. This result indicates an incipient, preclinical, systolic alteration, consistent with the results obtained in other obese pediatric cohorts (35, 36). Furthermore, GLS was significantly impaired among MUO patients compared to MHO, suggesting a negative effect of unfavorable metabolic profile on the early alteration of longitudinal myocardial deformation property. Moreover, GLS was significantly associated with HOMA-IR, and it was significantly impaired in OW and OB patients with insulin resistance. These findings let us speculate that insulin resistance is a strong independent predictor of subclinical LV dysfunction in obese, non-diabetic, children. Furthermore, insulin resistance has been demonstrated to have negative effects on myocardial function: a decreased myocardial glucose uptake, caused by a reduced availability of GLUT-4 sarcolemmal transporters, which results in a switch from aerobic glycolysis to a greater utilization of free fatty acids and in an increased oxidative stress and proinflammatory status (37); an activation of cardiomyocyte authopaghy, which causes loss of contractile cells (38); an increased deposition of the extracellular matrix and collagen associated with reduction of the degradation mechanisms (39).

GLS evaluation, in our cohort, assumes further importance and reliability in the assessment of the specific effect of obesity on myocardial contractility independent from diabetes and chronic arterial hypertension that could affect GLS (40, 41).

As previously reported (15, 42, 43), we also demonstrated a significant increase in both LV dimensions and LVM-index in OW and OB children compared to controls. These parameters were significantly affected by the severity of overweight, abdominal obesity, and insulin resistance. Precocious modifications of cardiac geometry could be determined, at least in part, by preload/afterload increase related to obesity (42).

### Diastolic Myocardial Function

Diastolic dysfunction has been associated with obesity both in adults as well as in children (44, 45). Accordingly, in our cohort, the E/A ratio was significantly lower in the OW and OB groups, and it was negatively significantly related to BMI SD, suggesting an incipient impaired myocardial relaxation of LV in these children. Moreover, the increased LVM-index and its significant association with BMI SD are expressions of the initial sign of obesity-related LV hypertrophy and early impaired myocardial relaxation (43). We also documented a significantly negative correlation between WC, LA strain, and E′, markers of diastolic dysfunction (46, 47). On the other hand, the E/E′ ratio as well as the LA volume, usually altered in the case of LV-elevated filling pressure in patients with chronic diastolic dysfunction, were included in the normal range, and they were not significantly different between groups. These findings are compatible with an early stage of LV dysfunction. Therefore, our results are consistent with the early signs of subclinical diastolic dysfunction significantly influenced by WC, BMI SD, and HOMA-IR.

### EAT Evaluation

EAT is a metabolically active adipose tissue strictly related to visceral and subcutaneous fat (19). The echocardiographic assessment of EAT is a sensitive and reliable marker of visceral adiposity (48). EAT and visceral fat exhibit similar pro-inflammatory cytokine mRNAs likely involved in chronic inflammation and potentially contributing to CVD pathogenesis (49). Therefore, EAT pathological increase seems to be involved in obesity-related CVD pathogenesis, probably promoted by EAT direct interaction with coronary vessels and myocardium, and mediated by paracrine and vasocrine secretion of pro-inflammatory cytokines and free fatty acids (20).

The amount of EAT in our patients was significantly higher among MUO patients, and it was strictly related to LV dimensions and mass, BMI SD, WC, and HOMA-IR; particularly, BMI SD was the stronger predictor of EAT amount. Data from the present series are consistent with the results of other pediatric studies, suggesting the possibility of a routine ultrasound assessment of EAT to estimate the CVD risk in obese children (50–52).

An interesting result of our study is the significant correlation between EAT and HOMA-IR, consistent with the reported data in adults (53, 54) and in contrast with the results of those few pediatric studies available (50, 55). In pediatric cohorts, despite not finding an association between EAT and HOMA-IR, EAT was able to identify patients with insulin resistance (19, 51). Linkage between EAT and insulin resistance may be explained by the findings of Fernadez-Trasancos et al. (56). These authors demonstrated, in EAT mesenchymal cells of patients with CVD, an association between a low adipogenic ability and insulin resistance, directly dependent from obesity, diabetes, and coronary artery disease. Insulin treatment rapidly improved adipogenic ability. Authors concluded that this association may contribute, at least partially, to the relation between EAT and CVD (56).

Based on our findings, we speculate that the accumulation of EAT may be promoted by the severity of overweight, abdominal obesity, and insulin resistance even in children.

### Arterial Evaluation

This study confirms a negative influence of childhood obesity on arterial structure and function. CIMT is considered a precocious non-invasive marker of atherosclerosis, strictly related to the severity of obesity and body fat distribution (57). In a large cross-sectional study, Hedblad et al. reported an increased CIMT in non-diabetic patients with insulin resistance (58). Arterial stiffness, able to reveal functional alteration, is a more sensitive parameter than intima–media thickness for the assessment of early vascular damage (59). An increased arterial stiffness, related to an increased CVD risk in adults (60), was documented also in obese pediatric cohorts, both in carotids (61, 62) as well as in aorta evaluation (63, 64), although, these findings were not univocally confirmed (65).

Incipient signs of vascular remodeling, increased carotids and AA stiffness have been documented in our obese population, especially among MUO patients. Moreover, BMI SD and WC showed a significant association with structural and functional indices of carotid and aorta, although an influence of SBP needs to be highlighted. Importantly, further studies are required to clarify the role of obesity, body fat distribution, dyslipidemia, insulin resistance, and other CV risk factors in atherosclerotic damage in children.

It might be argued that our study has some limitations. First, due to the cross-sectional design of the study, we are unable to verify the causal relationships between cardiometabolic risk variables and structural and functional myocardial modifications, which could be clarified in a longitudinal study involving a further enlarged cohort. Second, a 24-h blood pressure monitoring has not been performed in our patients.

In conclusion, severity of overweight, abdominal obesity, insulin resistance, and MUO phenotype negatively affect cardiovascular remodeling and subclinical myocardial dysfunction in OW and OB children. GLS and EAT are non-invasive and reliable indices that might be considered in echocardiographic evaluation to stratify cardiovascular risk in obese children and adolescents. MUO phenotype, characterized by higher prevalence of metabolic alterations and early cardiovascular modifications, is likely to increase the risk of developing cardiometabolic complications since the pediatric age. Therefore, a distinction between MHO and MUO phenotypes might be useful in planning a personalized follow-up approach in obese children, although it is needed before to establish univocal diagnostic criteria.

## Data Availability Statement

The datasets generated for this study are available on request to the corresponding author.

## Ethics Statement

The studies involving human participants were reviewed and approved by Ethics Commitee of Messina. Written informed consent to participate in this study was provided by the participants' legal guardian/next of kin.

## Author Contributions

DC and MW conceived the manuscript. DC, TA, and GP were involved in data collection. MC, LO, and LL performed the ultrasound examinations. DC, LL, and GBP selected and analyzed the references. AA carried out the data analysis. DC and LM prepared the tables. DC, LO, LL, and MW drafted and wrote the manuscript. All authors approved the submitted version of the manuscript.

### Conflict of Interest

The authors declare that the research was conducted in the absence of any commercial or financial relationships that could be construed as a potential conflict of interest. The reviewer LI declared a past co-authorship with one of the authors MW to the handling editor.
